# How *Varroa* Parasitism Affects the Immunological and Nutritional Status of the Honey Bee, *Apis mellifera*

**DOI:** 10.3390/insects3030601

**Published:** 2012-06-27

**Authors:** Katherine A. Aronstein, Eduardo Saldivar, Rodrigo Vega, Stephanie Westmiller, Angela E. Douglas

**Affiliations:** 1 Honey Bee Research Unit, USDA-ARS, Weslaco, TX 78596, USA; E-Mail: Ed.Saldivar@ars.usda.gov; 2 Department of Entomology, Cornell University, Ithaca, NY 14853, USA; E-Mails: rrv9@cornell.edu (R.V.); st342@cornell.edu (S.W.); aes326@cornell.edu (A.E.D.)

**Keywords:** *Apismellifera*, deformed wing virus, honey bee, nutritional response to parasite, *Varroa* mite

## Abstract

We investigated the effect of the parasitic mite *Varroadestructor* on the immunological and nutritional condition of honey bees, *Apis mellifera*, from the perspective of the individual bee and the colony. Pupae, newly-emerged adults and foraging adults were sampled from honey bee colonies at one site in S. Texas, USA. *Varroa*‑infested bees displayed elevated titer of Deformed Wing Virus (DWV), suggestive of depressed capacity to limit viral replication. Expression of genes coding three anti-microbial peptides (*defensin1, abaecin, hymenoptaecin*) was either not significantly different between *Varroa*-infested and uninfested bees or was significantly elevated in *Varroa*-infested bees, varying with sampling date and bee developmental age. The effect of *Varroa* on nutritional indices of the bees was complex, with protein, triglyceride, glycogen and sugar levels strongly influenced by life-stage of the bee and individual colony. Protein content was depressed and free amino acid content elevated in *Varroa*-infested pupae, suggesting that protein synthesis, and consequently growth, may be limited in these insects. No simple relationship between the values of nutritional and immune-related indices was observed, and colony-scale effects were indicated by the reduced weight of pupae in colonies with high *Varroa* abundance, irrespective of whether the individual pupa bore *Varroa*.

## 1. Introduction

It is widely accepted that the nutritional and immunological responses of animals to parasitization are not independent [1,2]. Both responses are demanding on nutritional reserves, such that an animal’s capacity to mount an immunological response can be critically dependent on its nutritional status [3,4], and parasite-mediated immunosuppression can have complex effects on the nutritional condition of its host [5]. Some effects appear to be mediated by a trade-off between the allocation of resources to immune function and nutritional reserves, or via changes to the animal’s regulatory network, especially the insulin/TOR signaling cascade, which links food intake to growth and metabolism and various aspects of immune function [6]. The detail of the “trialogue” between host nutrition and immune function and a parasitic infection, including the significance of the interaction between nutritional and immunological effects, is likely to vary widely among different host-parasite interactions.

The focus of this study is the interaction between the honey bee, *Apis mellifera*, and the mite *Varroa destructor* [7], an ectoparasite that feeds on the hemolymph of larvae, pupae and adult bees [8,9]. *Varroa* infestation is recognized as one of the most urgent problems facing the beekeeping industry [10,11,12]. There is evidence that *Varroa* mites depress both the nutritional condition and immunological capacity of honey bees [13,14]. Specifically, *Varroa*-infested bees have reduced weight and lifespan, elevated incidence of deformed wings and related phenotypes (shortened abdomen, miscoloration and paralysis) caused by high titers of the Deformed Wing Virus (DWV), and reduced immunological response to bacterial infection as quantified by expression levels of several antimicrobial peptides and immunity-related enzymes [15,16,17,18,19]. Nonetheless, the data are fragmentary and not entirely consistent. It is becoming increasingly apparent that an integrated understanding of *Varroa* effects requires analysis of the various indices in the same study populations of honey bees. 

The purpose of this study was to quantify the impact of experimental *Varroa* infestation on the immune response and nutritional status of honey bee colonies. The investigation was conducted on managed bee colonies in a nutritionally challenging habitat representative of commercial honey bee populations used in low intensity agricultural settings of southern Texas, USA. In this habitat, honey bee colonies are supported by the diverse flora with strong nectar flow in the spring, but they are vulnerable under the hot and dry conditions during summer and fall months that provide scarce nutritional resources. Focusing on colonies under the environmentally stressful conditions in August and September, we tested four hypotheses. (1) *Varroa*-infested bees have altered immunological status, as assessed by the expression of key immune-related genes, and DWV titer; (2) *Varroa*-infested bees are in poor nutritional condition, as indicated by reduced protein and amino acid content, and depressed reserves of lipid and glycogen; (3) The nutritional and immunological condition of the insects is correlated, such that the most nutritionally-deficient *Varroa*-infested insects are also the most immunologically-deficient; (4) Uninfested insects in colonies with high levels of *Varroa* infestation have depressed immunological and nutritional indices relative to colonies with low or no *Varroa* infestation, as a consequence of the poor overall condition of the colony. 

Our sampling strategy to test these hypotheses was dictated by the biology of the *Varroa* mites. Themites enter a brood cell (prior to capping of the cell), they attach to the larva and feed on the hemolymph continuously as the larva develops and pupates. The mites remain attached to their host as the newly emerged bee leaves the brood cell, and then they move among the worker bees in the hive, continuing to feed [8,9]. Our analysis focused principally on pupae and newly-emerged bees, to ensure that the scoring of insects as *Varroa*-infested or uninfested accurately reflected the infestation history of that insect. Insects that have borne *Varroa* since larvae and display clinical signs of DWV die or are removed from the colony as young adults. Foraging bees, in general, have lower levels of *Varroa* incomparison to brood and nurse bees [8,9]. Supplementary analyses of foraging bees were conducted to assess how the nutritional condition of these bees varies with the level of *Varroa* in the colony.

## 2. Experimental Section

### 2.1. The Insects and Sampling Procedure

The experimental colonies were derived from source colonies in TX apiaries that had been treated with Apiguard (thymol gel) to reduce *Varroa* population one year (2009) prior to this study. The experimental colonies were established in March 2010, using 10 three-pound packages of bees from source colonies along with a caged Kona Italian queens in each package. Packages were installed into new bee equipment (10 frame hive body, bottom board and migratory cover, 10 frames with the new wax coated plastic foundations). New colonies were placed in a new apiary and fed high-fructose corn syrup and MegaBee protein patties on *ad lib* basis. Kona queens were replaced by Koehnen Italian queens in April, 2010. Five experimental colonies were selected at random for *Varroa* infestation; a half frame of sealed brood comb infested with *Varroa* mites (14.5 mites per 100 bees) was introduced in May 2010. 

Samples were collected from the colonies on 13 July, 17 August and 8 September 2010: pink/purple-eyed pupae (n = 60), newly emerged adults (n = 72), and foragers (n = 72) as they entered the colony. With the exception of foragers, each insect was scored for the presence of *Varroa* mites and then transferred individually to a 1.5 mL vial and the fresh weight recorded ±1 μg accuracy. Samples were frozen and stored at −80 °C. In parallel, the level of *Varroa* infestation was determined using the alcohol shake method in samples of 280 bees per colony [20,21] and estimated as a number of mites per 100 adult bees. 

The indices of immunological nutritional status were conducted on bees collected on 17 August and 8 September. By this date in the season, three of the 10 colonies had died out, and sufficient bees for analysis were available from 6 of the remaining colonies for all assays except for the gene expression study, which was conducted on 5 colonies. 

### 2.2. Molecular Analysis

Total RNA was isolated from each bee sample using TRIzol® (Life Technologies, Carlsbad, CA, USA) following the manufacturer’s protocol. To test for residual DNA contamination, 1 μL RNA was used as template in PCR, using primers that amplify a *defensin 1* gene fragment that includes a 286 bp intron. Samples that tested positive for the presence of the intron were treated with DNA-free™ Kit (Ambion-Life Technologies, Carlsbad, CA, USA) until DNA was not detectable. Poly-A RNA was purified from the total RNA samples using MicroPoly(A)Purist™ (Ambion-Life Technologies, Carlsbad, CA, USA) following manufacturer’s protocol, and its purity was checked on a 1.0% denaturing agarose/formaldehyde gel before quantification using an Eppendorf BioPhotometer. Double-stranded cDNA was synthesized with 500 ng poly-A RNA template, SuperScript™ Double-Stranded cDNA Synthesis Kit (Invitrogen-Life Technologies, Carlsbad, CA, USA), and oligo-dT primers according to the manufacturer’s protocol.

The expression levels of *defensin1*, *abaecin*, *hymenoptaecin, vitellogenin* and *hexamerin 70b*, and the abundance of DWV [13] were determined by qRT-PCR normalized to *ß-actin* using CFX96™ Real-Time PCR Detection System (BioRad, Inc.), following evidence that this gene is stably expressed and the reference gene of choice [22,23]. Amplification was performed in 10 μL reaction volumes, containing 0.5 U of GoTaqR Flexi DNA polymerase (Promega Co., Madison, WI, USA) with the colorless 5× GoTaqR Flexi buffer, 0.38 mM dNTP mix, 5.0 mM MgCl_2_, 0.35 μM of each primer (Table 1), 0.33 μL SYBR-Green (Invitrogen., 1/1,000 stock dilution), and 1 μL cDNA. Reactions were run for 40 cycles at 95 °C (5 s) and 60 °C (30 s) after initial denaturing at 95 °C for 3 minutes, and with a final extension of 72 °C (2 min) followed by a melt curve analysis. All experiments included two technical replicates of three biological replicates (for each developmental stage, colony and *Varroa*-category) andtemplate-free controls.The qRT-PCR data was analyzed using the comparative delta delta C_t_ method, with C_t_ values normalized to ß-actin. Fold change in expression of the target genes was quantified as by Pfaffl [24], using the equations for relative standard curves and relative efficiency plots calculated by GraphPad Prism software (GraphPad Software, Inc., La Jolla, CA, USA). 

**Table 1:** List of PCR primers.

Primer names and GenBank Accession [#]	Primers 5′→3′
Deformed wing virus [AB605386] DWV_F DWV_R	GACAAAATGACGAGGAGATTGTT ACTACCTGTAATGTCGTCGTGTT
*Defensin 1 *[U15955] Def1_F Def1_R	GTTGAGGATGAATTCGAGCC TTAACCGAAACGTTTGTCCC
*Abaecin* [U159554] Abaecin_F Abaecin_R	GGTAGTGATATTTATCTTCGC TTGAGGCCATTTAATTTTCGG
*Hymenoptaecin* [U15956] Hymenoptaecin_F Hymenoptaecin_R	ATGGATTATATCCCGACTCG TCTAAATCCACCATTTATTCC
*Hexamerin70b *[NM_001011600] Hex70b_F Hex70b_R	CCGCTCTTCAAATGTGGTCTAC GATAGGTAAAAGGTTTGTGGTTC
*Vitellogenin* [NM_001011578] Vg_FVg_R	TGGCTCTGGACGGTGAAC CCCTCGCATTGGTTACTGAT
*Actin* [AB023025] Actin_F Actin_R	TGAAGTGCGATGTCGATATC GAGATCCACATCTGTTGGAA

### 2.3. Nutritional Indices

Each pupa or bee was thawed on ice, and then homogenized in 1 mL ice-cold buffer comprising 10 mM Tris, 1 mM EDTA pH 8.0 and 0.1% (v/v) Triton-X-100 with hand-held homogenizer, and centrifuged at 7,000 g at 4 °C for 10 min. The supernatant was used for analysis of protein, triglyceride and carbohydrates using coupled colorimetric assays with an xMark^TM^ microplate spectrophotometer, following manufacturer’s instructions (5 replicates per assay). The assay kits were the triglyceride assay kit of Sigma (catalogue number TG-5-RB); the Coomassie Brilliant Blue microassay method of BioRad (catalogue number 500-0201), with bovine serum albumin as standard (1.25–25 mg protein mL^−1^)for protein; and the glucose assay kit of Sigma (catalogue number GAGO20) for glucose and, following trehalase (3.7 U mL^−1^) and amyloglucosidase (2 U mL^−1^) treatment, for trehalose and glycogen, respectively. The sugar analyses (glucose and trehalose) were restricted to pupae because datasets for adult bees were confounded by endogenous trehalase activity and sugars in the gut contents of some individuals. 

For the amino acid analysis, 1 μL supernatant was brought to 20 μL in PBS, incubated on ice for 30 min with 20 μL 40 mM HCl, and centrifuged at 18,000 g for 15 min at 4 °C. The supernatant was filtered through a 0.45 μm filter plate (Millipore) by centrifugation at 1,500 g for 10 min, and 5 μL filtrate was derivatized with AccQ Tag (Waters), following manufacturer’s protocol, and injected into Waters Acquity UPLC with PDA detector and AccQ-Tag Ultra 2.1 × 100 mm column. The gradient was: 0–10 min, 99% A; 10–35 min, linear gradient to 65% A; 35–40 min, 65% A; 40–42 min, linear gradient to 100% B, where A is 90% AccQ-Taq Ultra Eluent in water, and B is Accq-Taq Ultra Eluent B. Amino acids were determined by comparison to standards, 1–100 pmol protein-amino acids μL^−1^ (Waters amino acid hydrolysate standard #088122, supplemented with asparagine, tryptophan and glutamine).

### 2.4. Statistical Analysis

All data were checked for normality by the Anderson-Darling test and homogeneity of variance by Levene’s test, and data that did not meet these criteria were analyzed by non-parametric methods. The abundance of *Varroa* in uninfested colonies and colonies experimentally infested with *Varroa* was compared by the MannWhitney U test for each of the four test months (July-September). The fold-change of gene expression in *Varroa*-infested bees relative to uninfested bees was compared to the null hypothesis of no change by t-tests, with Bonferroni correction for multiple tests.Preliminary analysis revealed no significant correlations between nutritional indices obtained for either pupae or hive bees in September (Pearson’s correlation analysis). Therefore, the insect weight and nutritional density data were analyzed by individual ANOVA tests, with three main factors: Vbee (whether the insect bore *Varroa*); Vcolony (whether the *Varroa* abundance was above the recommended treatment threshold of 15 mites per 100 bees); and individual Colony nested within Vcolony. The principal components analysis of free amino acids in the pupae and hive bees used a correlation matrix to standardize variables, with axes selected to display separation of *Varroa*-infested and uninfested bees. Linear regression analysis was used to investigate the relationship between nutritional indices of uninfested bees and *Varroa* abundance in the same colony, and the relationship between immune-related indices and nutritional indices.

## 3. Results

### 3.1. Abundance of Varroa in the Honey Bee Colonies

**Figure 1:**
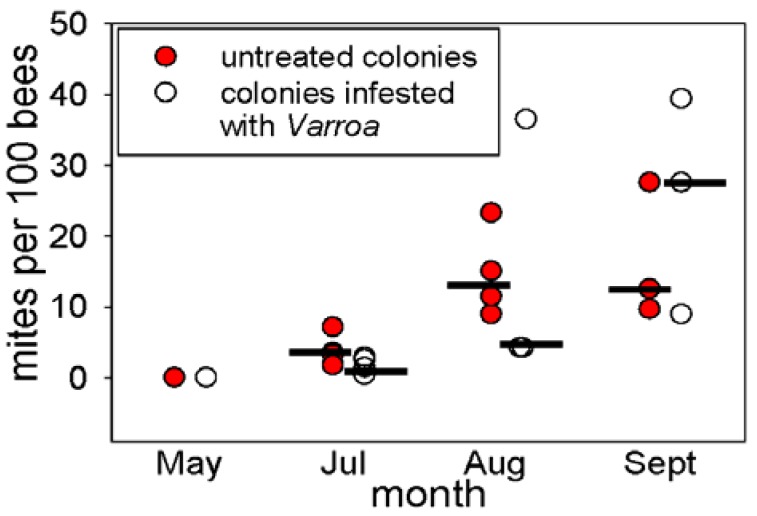
The abundance of *Varroa* in the honey bee colonies (median shown as black bar). The economic threshold for intervention is 15 mites per 100 bees.

*Varroa* was not detected in the mitocide-treated colonies set up in May 2010, but was present in all colonies tested in July, August and September, 2010. The median abundance of *Varroa* was significantly higher in untreated colonies than experimentally-infested colonies in July (Mann Whitney U: W = 38, p < 0.05), but did not differ between the two colony types in August (W = 18, p = 0.59) or September (W = 14.5, p = 0.72) (Figure 1). 

### 3.2. Effect of Varroa on DWV Titer and Immune-Related Indices of Bees

**Figure 2:**
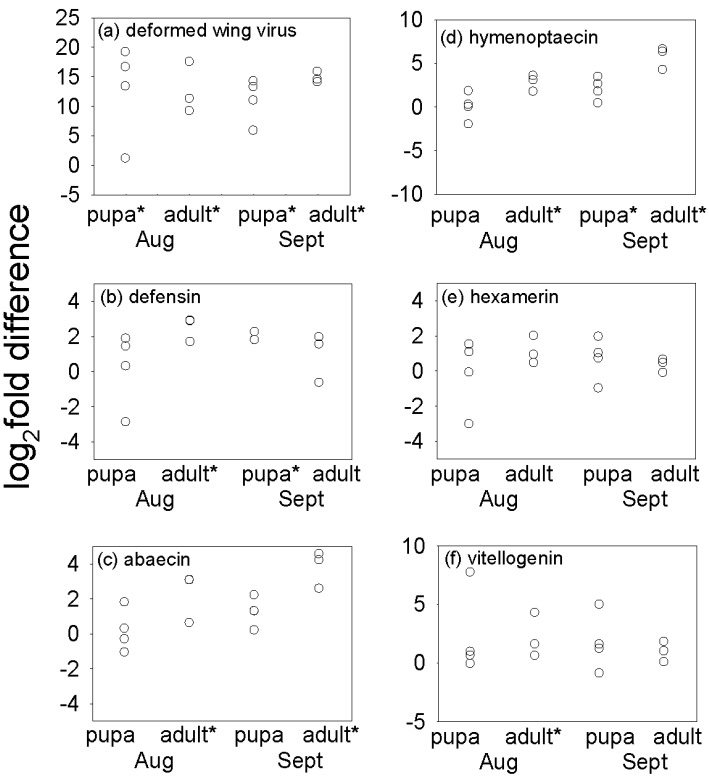
Impact of *Varroa* infestation of pupae and newly-emerged adults on (**a**) the titer of deformed wing virus; expression of anti-microbial peptides: *defensin1* (**b**), *abaecin* (**c**) and *hymenoptaecin* (**d**); and expression of storage proteins: *hexamerin 70b* (**e**) and *vitellogenin* (**f**). Mean values of log_2 _(*Varroa*-infested/uninfested)of three replicate samples from each colony are shown, and values significantly different from zero (after Bonferroni correction for four tests) are indicated *. Full statistical analysis is shown in Supplementary Table S1.

The titer of Deformed Wing Virus (DWV) in pupae and newly-emerged bees was quantified in August and September, when there were sufficient *Varroa*-infested insects for analysis. DWV was detected in all samples, and the DWV titer was significantly elevated in *Varroa-*infested insects, relative to uninfested pupae and newly-emerged bees (Figure 2a, Supplementary Table S1), with differences ranging from less than two-fold to >300,000-fold. Significant among-colony variation in the ratio of DWV titer in infested/uninfested insects was obtained for pupae in August (F_4,14_ = 21.90, p < 0.001) but not in pupae in September or in newly-emerged bees. Foragers in general bore a very low titer of the virus (data not shown). 

Our approach to investigate further the impact of *Varroa* on the immune status of the honey bees was to quantify the expression level of three antimicrobial peptide (AMP) genes, *defensin1*, *abaecin* and *hymenoptaecin* in pupae and newly-emerged adults sampled in August and September. Individuals bearing *Varroa* had significantly higher transcript abundance of these immune-related genes for at least two of the four datasets, with the mean fold-difference varying between two-fold and >30-fold (Figure 2b–d, Supplementary Table S1). No instance of a significant reduction in gene expression was identified in *Varroa*-infested bees. Across the full dataset, the expression levels did not differ between colonies with *Varroa* infestation above or below the threshold (15 mites per 100 bees) for intervention (p > 0.05) and significant among-colony variation was restricted to two AMPs, *abaecin* and *defensin1* in August pupae (F_4,14_ = 10.06, p = 0.002; F_4,14_ = 21.90, p < 0.001). 

The relationship between AMP expression and DWV titer was investigated by correlation analysis. The log_2 _fold difference in DWV titer between *Varroa*-infested and uninfested pupaewas significantly correlated with the log_2_-fold-difference in transcript abundance of *defensin1*, but not *abaecin* or *hymenoptaecin* in August (r = 0.998, p < 0.001). No significant correlations were identified in newly-emerged bees in August or September, or for pupae in September. 

### 3.3. Effect of Varroa on Nutritional Status of Bees

**Figure 3:**
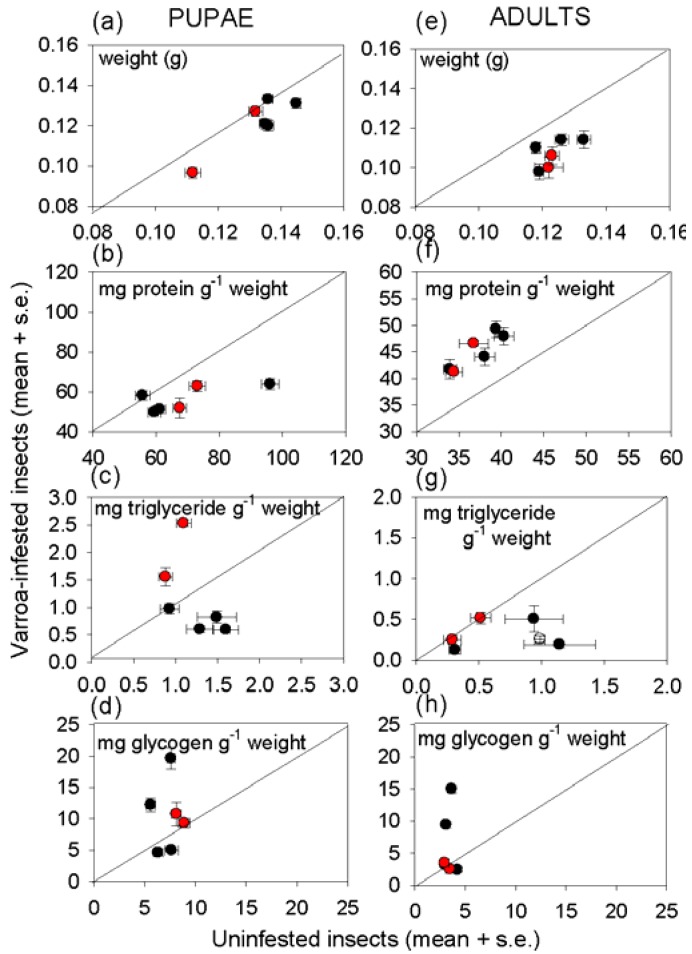
The weight and nutritional indices of uninfested and *Varroa*-infestedinsects (mean ± s.e, 12 replicates); with the diagonal line of equivalence; (**a-d**) showing nutritional conditions of pupae; and (**e-h**) newly emerged adult bees. Data for colonies with *Varroa* above the threshold for intervention are shown in red, and colonies below the threshold in black. The statistical analysis is shown in Supplementary Table S2.

The weight of pupae and hive bees bearing *Varroa* was significantly depressed relative to *Varroa*-free individuals (Figure 3a,e, Supplementary Table S2a). For pupae, but not newly-emerged adults, mean weight was also depressed in colonies with high (*i.e.*, above treatment threshold) *Varroa* abundance, irrespective of whether the individual bore *Varroa*, and the magnitude of this effect varied with colony.

The nutritional condition of the insects was significantly affected by *Varroa* (Figure 3, Supplementary Table S2), although the effects differed between pupae and newly emerged bees. The protein density (*i.e.*, protein content per unit weight) was significantly depressed in *Varroa*-infested pupae, but elevated in *Varroa*-infested hive bees (Figure 3b,f), although this was not generally reflected in the expression levels of two key storage protein genes, *hexamerin70b* and *vitellogenin* (Figure 2e,f). The effect of *Varroa* infestation on triglyceride density varied with the *Varroa* status of the colony, being elevated in *Varroa*-infestedpupae (but not newly emerged adult bees) from colonies with high *Varroa* infestation, and reduced in *Varroa*-infested adult bees (but not pupae) from colonies with low *Varroa* infestation (Figure 3c,g).

The single most consistent determinant of the nutritional indices was the colony from which the insects were sampled; every nutritional index scored varied significantly with colony or the interaction between colony and whether the insect bore *Varroa* (Supplementary Table S2). Colony effects were particularly marked for glycogen density, which was significantly elevated in pupae and newly-emerged bees bearing *Varroa* in two of the four colonies (colonies 1 and 4) with low *Varroa* abundance (Figure 3d,h). 

**Figure 4:**
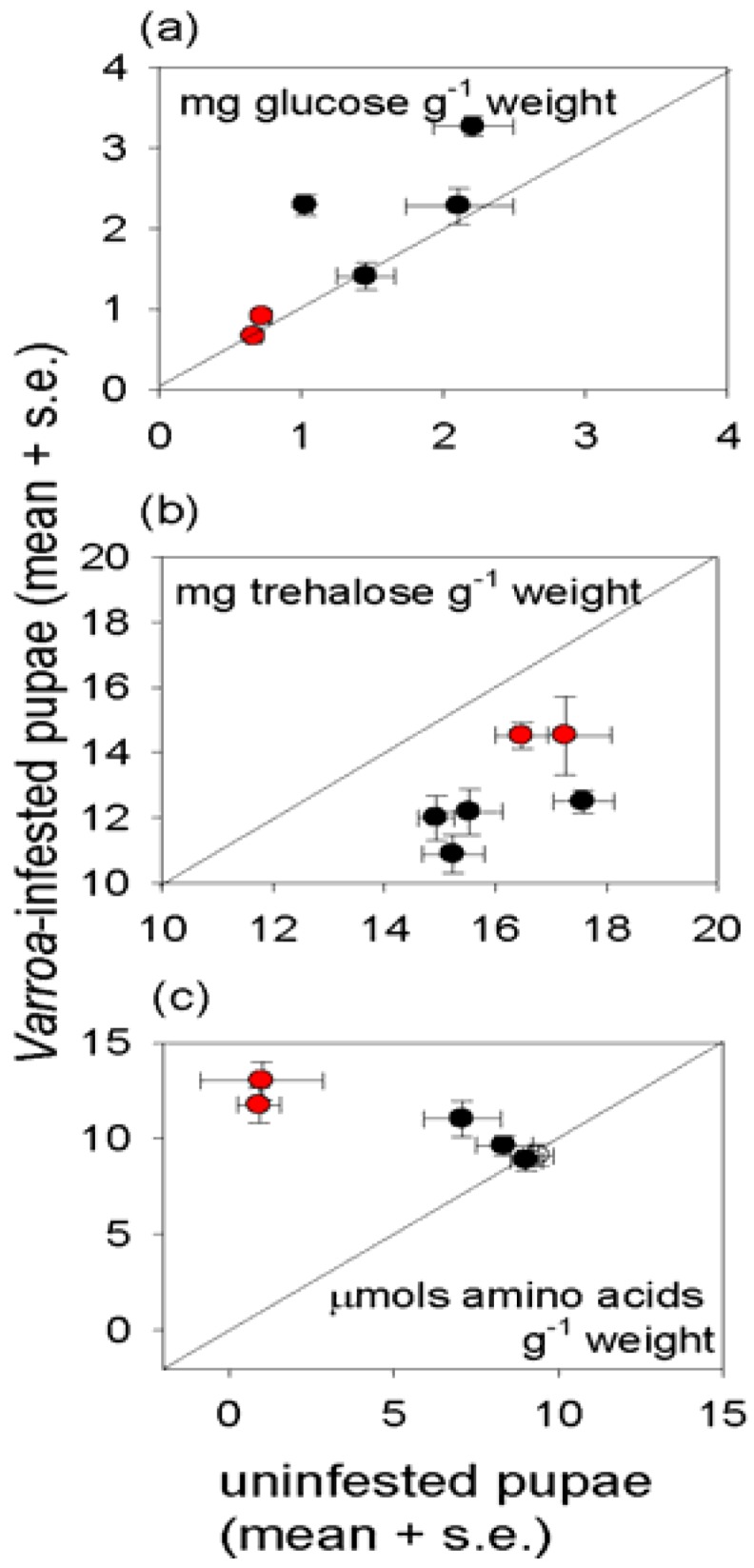
Free metabolites in uninfested and *Varroa*-infestedhoney bees (mean ± s.e, 12 replicates), with the diagonal line of equivalence. Data for colonies with *Varroa* above the threshold for intervention are shown in red, and colonies below the threshold in black. The statistical analysis is shown in Supplementary Table S2.

The metabolite analysis focused on two key sugars, glucose and trehalose, and the 20 amino acids that contribute to protein (Figure 4a–c). Individual colony effects were important for the sugars. *Varroa*-infestedpupae had significantly reduced trehalose density (Figure 4b), especially in colonies with low *Varroa* abundance; and the glucose density (Figure 4a) was significantly elevated in pupae bearing *Varroa* in two of the four colonies with low *Varroa* abundance (colonies 1 and 4: the same colonies with elevated glycogen contents). The total amino acid density of pupae was significantly elevated by *Varroa* infestation, at the level of both the individual insect and colony (Figure 4c); but no significant effect was obtained for newly-emerged adults (Supplementary Table S2). 

**Figure 5:**
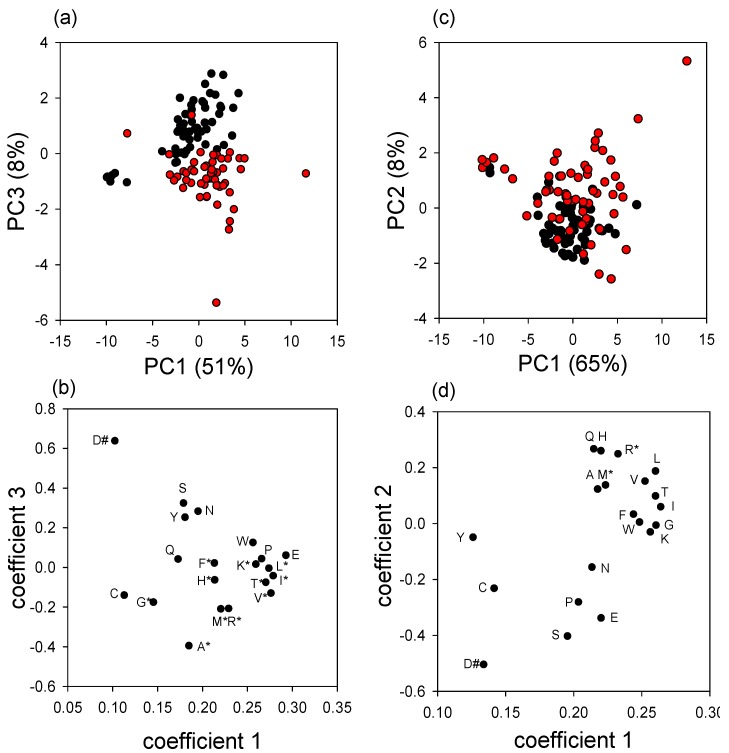
Principal components analysis of the free amino acid content of the honey bees (with open symbols uninfested and closed symbols *Varroa*-infested insects). Data for colonies with *Varroa* above the threshold for intervention are shown in red, and colonies below the threshold in black. (**a**) principal components 1 and 3 of pupae; (**b**) coefficients for pupae; (**c**) principal components 1 and 2 of newly-emerged bees; (**d**) coefficients for newly-emerged bees [#significantly elevated in uninfested insects; * significantly elevated in *Varroa*-infested insects].

Principal components analysis of the composition of the free amino acid pools of the insects (Supplementary Table S3) yielded near-separation along two axes between *Varroa*-infested and *Varroa*-free insects, for both pupae (Figure 5a) and newly-emerged adults (Figure 5c), accounting for 58% and 72% of the variance, respectively. Individual ANOVAs (with Bonferroni correction for 20 tests) (Supplementary Table S3) confirmed the inspection of the PC coefficients (Figure 5b) that a set of 11 AAs contributed to the elevated FAA titers in *Varroa*-infested pupae. This set includes 9 of the 10 essential amino acids (all but tryptophan). Exceptionally, aspartate was significantly depressed in *Varroa*-infested pupae. The amino acid titers of newly-emerged adult bees displayed a similar trend, although with weaker separation of *Varroa*-infested and uninfested bees and significant elevated titers of just two amino acids, arginine and methionine (Figure 5d, Supplementary Table S3). 

To investigate the effect of *Varroa* abundance on the nutritional condition of the bees further, the nutritional indices for uninfested pupae, newly-emerged bees and foragers was regressed on *Varroa* abundance quantified in the same month and, for August and September, in the previous month. None of the regressions were statistically significant, indicating that the level of *Varroa* infestation in the colony does not have a consistent effect on any tested nutritional index of the bees. The relationship between nutritional indices and immune-related indices (DWV titer and AMP transcript abundance) was also investigated by correlation analysis: no significant correlation between any nutritional and immune-related index was identified.

## 4. Discussion and Conclusions

An important factor contributing to the threat posed by *Varroa* to honey bee populations is the high incidence of among-colony transmission due to the drifting bees (*i.e.*, foraging bees that return to the “wrong” colony) [25]. The capacity for rapid among-colony transmission of *Varroa* is illustrated by this study, in which five initially mite-free colonies had acquired *Varroa* within 8 weeks, despite no experimental introduction of *Varroa*. The source(s) of the *Varroa* in these colonies was not investigated, but could have included foraging bees from the nearby experimentally-infested colonies or feral bees. The incidence of *Varroa* in feral honey bees is high in the area of S. Texas, USA, used in this study. In other studies in the same region, untreated honey bee colonies have collapsed within five months, apparently due to *Varroa* infestation [26]. Together with the finding here that the abundance of *Varroa* in the untreated colonies was significantly higher than in the experimentally-infested colonies at the first time of assay (Figure 1), these consideration suggest that the *Varroa* in the colonies were likely predominantly of environmental origin.

Consistent with previous reports [13,14,15,17,27], DWV titer was significantly elevated in *Varroa*-infested insects, a result that has been interpreted previously [13] to indicate that *Varroa* may reduce the immunological capacity of the honey bee to limit replication of DWV. For example, honey bee defenses based on RNAi may be impaired in *Varroa-*infested bees. Intriguingly, expression of the autophagic-specific gene 18 (*Atg18*) is reduced in *Varroa*-infested bees [28]. This could be indicative that elements of the defensive response against DWV are intact because the autophagic vacuole can act as a site of replication for certain RNA viruses, e.g., dengue virus, in insects [29,30]. Alternatively, activation of virus replication in *Varroa* infested bees has been discussed by [27,31]. Moreover, the co-infection of DWV with another single-stranded RNA virus, *Varroa destructor*-1 virus (VDV-1) and/or their recombinants were implicated in facilitating virus transmission and replication in the host [32,33].

We had predicted that *Varroa*-infested bees would display reduced expression of the AMP genes *defensin1*, *abaecin* and *hymenoptaecin* (see Introduction), based on published evidence that the expression of these genes in newly-emerged bees challenged with *E. coli *was elevated to a greater extent in uninfested bees than *Varroa*-infested bees [13]. Contrary to our prediction, expression of these AMP genes was not reduced by *Varroa* infestation in this study (Figure 2). Our results are, however, consistent with one report of no significant changes in expression of immune-related genes in *Varroa* parasitized bees [28]. Taken together, these studies suggest that the impact of *Varroa* on AMP expression may be variable, being influenced for example by honey bee genotype and environmental conditions.

*Varroa*-infested honey bees are profoundly unhealthy, as indicated by their lower weight and reduced density of multiple nutrients, relative to uninfested bees of the same developmental stage (Figure 3, Figure 4).Nevertheless, the impact of *Varroa* on honey bee nutrition is complex, and no individual index of nutritional condition could be related to any individual index of immunological condition of the honey bees tested in this study. Despite the evidence for a relationship between immune function and nutritional condition in insects and other animals (see Introduction), attempts to identify the patterns of interaction can easily be confounded by other factors, especially in field populations as studied here.

The protein content of the insects illustrates the complexity of nutritional responses to *Varroa*. Reduced protein density of *Varroa*-infested bees is predicted frompublished reports that *Varroa* depresses the protein content of newly-emerged adult bees [34] and the abundance of transcripts functioning in protein metabolism [35]. This expectation was confirmed for pupae, but not newly-emerged adults in this study. *Varroa-*infested pupae also had significantly elevated free amino acid content, suggesting that protein synthesis, and ultimately growth, is inhibited by factors other than the supply of amino acids. The high essential amino acid content of the free amino acids in *Varroa*-infested pupae would be particularly nutritious for the mites, raising the possibility that components in the *Varroa* saliva interfere with protein and amino acid nutrition of the honey bee to promote mite nutrition. The elevated protein content of infested adult bees suggests that these candidate effects of *Varroa* on honey bee metabolism may be stage-dependent. 

The importance of extraneous uncontrolled factors in determining the nutritional condition of honey bees is illustrated by pervasive and profound effect of colony on insect nutritional condition, especially with respect to energy and carbon allocation. For triglyceride, this effect could be linked, at least partly, to the level of *Varroa* infestation of the colony. Specifically, the high triglyceride density in pupae and newly-emerged bees from colonies with above-threshold *Varroa* incidence (Figure 3) may reflect the impaired nutritional input to larvae, such that offspring with elevated triglyceride levels have preferential survival rates. Additionally or alternatively, offspring may respond to poor nutritional conditions by preferential allocation to triglyceride. Further research is required to investigate these possibilities because previous studies of larval malnourishment, reviewed in [36], have not addressed effects of *Varroa* infestation on nutritional condition of the honey bee. Glycogen and glucose densities also varied among colonies, irrespective of *Varroa* incidence. The multiple contributory factors may include micro-environmental variation in abiotic conditions experienced by different colonies, proximity to different food sources, and the incidence of parasites and pathogens other than *Varroa*. 

The immune system function of the honey bee has evolved in the context of eusociality, which both increases the potential for rapid within-colony spread of pathogens and parasites, and provides the opportunity for colony-level defenses against infection [37,38]. An important element to the design of this study was the use of multiple colonies, enabling us to discriminate colony-level effects on the honey bee response to *Varroa*. The low weight of pupae in colonies with high *Varroa* abundance, irrespective of whether the individual pupa was infested, provides a firm indication that *Varroa* can deleteriously affect larval provisioning at the colony scale. Other colony-scale effects were generally not evident. A larger number of colonies across multiple sites would provide a stronger test of hypothesized colony-level effects on individual nutritional and immune-related indices than was possible with this study. The key conclusion of this study is, consequently, the pervasive deleterious effect of *Varroa* on the immunological response and nutritional condition of honey bees in an environmentally-challenging habitat.

## Supplementary Files

Supplementary File 1
