# Supplementary material for: How Varroa Parasitism Affects the Immunological and Nutritional Status of the Honey Bee, Apis mellifera
Source: Insects. 2012 Jun 27;3(3):601–15. doi: 10.3390/insects3030601 (PMC4553578; doi:10.3390/insects3030601)
Supplement: Supplementary File 1 [file insects-03-00601-s001.pdf]

Supplementary Materials

Table S1. Statistical analysis of DWV titers and gene expression levels. The fold difference in RNA of interest between *Varroa*-infested and uninfested bees was $\log_2$-transformed and compared to the null hypothesis of $\log_2$ (fold difference) = 0 (no difference) by t-test, with Bonferroni correction for four tests for each gene. For this analysis, samples from all colonies were pooled, following confirmation of no significant among-colony variation ($p > 0.05$): August pupae, n = 15; August newly emerged bees, n = 12; September pupae, n = 12; September newly-emerged adults, n = 9. NS – not significant, * $0.0125 > p > 0.001$; ** $p < 0.001$

(a) DWV titer

Sample	t	p
August pupae	7.306	**
August hive bees	8.670	**
September pupae	6.799	**
September hive bees	11.983	**

(b) hexamerin

Sample	T	p
August pupae	0.789	NS
August hive bees	2.321	NS
September pupae	1.4628	NS
September hive bees	1.328	NS

(c) vitellogenin

Sample	T	p
August pupae	2.947	NS
August hive bees	4.309	*
September pupae	1.5739	NS
September hive bees	0.919	NS

(d) hymenoptaecin

Sample	T	p
August pupae	1.365	NS
August hive bees	4.877	**
September pupae	11.152	**
September hive bees	6.146	**

(e) defensin

Sample	T	p
August pupae	0.495	NS
August hive bees	3.366	*
September pupae	10.481	**
September hive bees	1.109	NS

Table S1. *Cont.*

(f) abaecin

Sample	T	p
August pupae	1.763	NS
August hive bees	4.119	*
September pupae	7.627	**
September hive bees	5.310	**

Table S2. Statistical analysis of the nutritional indices and metabolites in pupae and hive bees (Vbee=uninfested vs *Varroa*-infested insect; Vcolony=*Varroa* prevalence above or below the treatment threshold; colony(Vcolony)=colony nested within Vcolony. The critical probability for indices quantified for both pupae and hive bees is 0.025, following Bonferroni correction for two tests. [* p<0.05 or <0.025; ** p<0.01; *** p<0.001].

a. Weight

Source of variance	df	Pupae[1]	Hive bees[2]
Vbee	1	53.38***	73.92***
Vcolony	1	102.47***	3.32
Colony(Vcolony)	4	31.15***	6.56***
Vbee x Vcolony	1	0.09	1.06
Vbee*colony(Vcolony)	4	3.69	1.47

[1] error df =124; [2] error df = 132

b. Protein density

Source of variance	df	Pupae[1]	Hive bees[2]
Vbee	1	46.47***	47.50***
Vcolony	1	1.06	3.08
Colony(Vcolony)	4	33.94***	4.81**
Vbee x Vcolony	1	0.03	0.05
Vbee*colony(Vcolony)	4	11.74***	0.41

[1] error df =124; [2] error df = 132

c. Triglyceride density

Source of variance	df	Pupae[1]	Hive bees[2]
Vbee	1	5.78**	7.55**
Vcolony	1	23.68***	2.33
Colony(Vcolony)	4	3.93**	3.23*
Vbee x Vcolony	1	69.44***	6.59*
Vbee*colony(Vcolony)	4	5.11**	1.50

[1] error df =124; [2] error df = 125

Table S2. *Cont.*

d. Glycogen density

Source of variance	df	Pupae[1]	Hive bees[2]
Vbee	1	14.85***	2.05
Vcolony	1	1.09	3.36
Colony(Vcolony)	4	23.21***	2.87*
Vbee x Vcolony	1	2.48	2.63
Vbee*colony(Vcolony)	4	21.49***	3.02*

[1] error df =124; [2] error df = 132

e. Glucose content

Source of variance	df	Pupae[1]
Vbee	1	6.75*
Vcolony	1	89.37***
Colony(Vcolony)	4	14.57***
Vbee x Vcolony	1	3.76
Vbee*colony(Vcolony)	4	4.44**

[1] error df =121

f. Trehalose content

Source of variance	Df	Pupae[1]
Vbee	1	58.00***
Vcolony	1	19.86***
Colony(Vcolony)	4	3.29*
Vbee x Vcolony	1	3.69
Vbee*colony(Vcolony)	4	1.13

[1] error df =121

g. Free amino acid content

Source of variance	Df	Pupae[1]	Hive bees[2]
Vbee	1	13.96***	0.13
Vcolony	1	6.91**	2.74
Colony(Vcolony)	4	0.03	0.35
Vbee x Vcolony	1	3.22	0.09
Vbee*colony(Vcolony)	4	2.14	2.45

[1] error df = 102; [2] error df = 108

Table S3. Amino acid content of pupae and hive bees from six colonies (#1, #4 etc.)

Amino acid	Concentration (nmol mg^{-1}weight) Mean ± s.e. (10 replicates)*											
	Uninfested insects						*Varroa*-infested insects					
	#1	#4	#5	#6	#7	#8	#1	#4	#5	#6	#7*	#8
Pupae												
Alanine	934 ± 129	830 ± 56	782 ± 84	728 ± 127	784 ± 180	1148 ± 134	1083 ± 92	1165 ± 153	1115 ± 94	1491 ± 129	1822 ± 207	2273 ± 561
Arginine	517 ± 29	816 ± 53	570 ± 80	626 ± 105	219 ± 89	761 ± 42	768 ± 62	992 ± 54	958 ± 51	1081 ± 109	778 ± 71	819 ± 94
Aspartate	108 ± 10	96 ± 9	96 ± 17	84 ± 14	81 ± 20	66 ± 8	38 ± 11	42 ± 5	50 ± 8	37 ± 6	53 ± 5	35 ± 4
Asparagine	991 ± 59	739 ± 61	645 ± 86	628 ± 112	887 ± 197	988 ± 79	878 ± 82	519 ± 57	519 ± 36	682 ± 107	1155 ± 68	885 ± 101
Cysteine	16 ± 5	4 ± 1	8 ± 2	8 ± 1	11 ± 3	8 ± 2	16 ± 3	15 ± 1	19 ± 2	14 ± 2	13 ± 2	18 ± 8
Glutamate	1101 ± 63	1105 ± 70	1015 ± 126	890 ± 151	872 ± 191	1180 ± 95	1115 ± 90	1075 ± 77	1147 ± 83	1400 ± 168	1338 ± 85	1292 ± 133
Glutamine	2206 ± 170	1366 ± 93	1717 ± 188	1468 ± 325	2644 ± 670	2064 ± 240	1828 ± 111	1574 ± 148	1646 ± 91	2037 ± 223	2785 ± 313	3380 ± 586
Glycine	1644 ± 191	1118 ± 156	1168 ± 218	815 ± 168	1528 ± 381	2030 ± 299	1890 ± 212	1061 ± 85	1052 ± 54	1420 ± 184	2973 ± 438	2914 ± 333
Histidine	310 ± 23	238 ± 18	243 ± 25	235 ± 47	348 ± 72	356 ± 29	339 ± 19	238 ± 12	258 ± 14	349 ± 25	545 ± 52	424 ± 46
Isoleucine	437 ± 58	577 ± 48	520 ± 69	462 ± 78	416 ± 89	540 ± 35	526 ± 38	717 ± 51	762 ± 45	789 ± 80	659 ± 37	519 ± 63
Leucine	708 ± 51	833 ± 65	752 ± 106	691 ± 117	575 ± 127	720 ± 49	768 ± 60	1011 ± 68	1111 ± 72	1179 ± 123	851 ± 80	695 ± 91
Lysine	695 ± 62	821 ± 83	657 ± 118	694 ± 133	448 ± 103	605 ± 51	681 ± 83	946 ± 110	996 ± 67	1126 ± 154	477 ± 52	718 ± 84
Methionine	65 ± 6	100 ± 10	90 ± 14	70 ± 10	53 ± 10	70 ± 7	65 ± 5	131 ± 13	144 ± 16	135 ± 19	102 ± 14	86 ± 13
Phenylalanine	283 ± 18	492 ± 51	469 ± 76	342 ± 52	294 ± 65	309 ± 23	317 ± 34	601 ± 30	682 ± 48	586 ± 60	452 ± 48	284 ± 43
Proline	2945 ± 164	2572 ± 152	2384 ± 262	2439 ± 430	2307 ± 519	3032 ± 238	2692 ± 211	2199 ± 137	2750 ± 194	3266 ± 328	3770 ± 398	3315 ± 393
Serine	3002 ± 371	3127 ± 162	3175 ± 347	2002 ± 348	3177 ± 722	3200 ± 229	3160 ± 247	2583 ± 266	285 ± 228	3012 ± 339	4269 ± 129	3609 ± 387
Threonine	191 ± 42	415 ± 25	345 ± 51	327 ± 57	356 ± 78	395 ± 31	317 ± 27	481 ± 38	468 ± 37	517 ± 54	466 ± 58	447 ± 46
Tryptophan	306 ± 37	363 ± 15	349 ± 33	299 ± 49	303 ± 65	352 ± 11	351 ± 21	392 ± 12	425 ± 18	423 ± 27	392 ± 47	307 ± 36
Tyrosine	1695 ± 110	1884 ± 285	1196 ± 212	895 ± 136	913 ± 257	671 ± 55	747 ± 64	1351 ± 68	1691 ± 189	1765 ± 313	1937 ± 361	616 ± 79
Valine	617 ± 43	572 ± 43	529 ± 55	457 ± 79	527 ± 122	673 ± 76	641 ± 49	659 ± 48	656 ± 40	757 ± 62	946 ± 82	864 ± 95

Table 3. *Cont.*

Newly-emerged adults												
Alanine	975 ± 183	551 ± 185	907 ± 58	870 ± 89	995 ± 81	1053 ± 72	1080 ± 120	846 ± 139	946 ± 124	1323 ± 146	1352 ± 249	1212 ± 165
Arginine	496 ± 103	611 ± 34	435 ± 34	459 ± 22	376 ± 36	547 ± 32	895 ± 79	638 ± 100	726 ± 100	762 ± 59	714 ± 156	814 ± 110
Aspartate	112 ± 20	156 ± 21	111 ± 8	130 ± 15	152 ± 11	152 ± 10	63 ± 4	64 ± 13	101 ± 23	160 ± 27	77 ± 13	126 ± 25
Asparagine	347 ± 63	375 ± 56	360 ± 27	280 ± 24	436 ± 28	375 ± 26	322 ± 45	253 ± 39	233 ± 29	326 ± 24	337 ± 53	397 ± 61
Cysteine	9 ± 2	9 ± 1	12 ± 1	11 ± 1	9 ± 1	12 ± 1	16 ± 3	8 ± 1	18 ± 2	17 ± 2	12 ± 2	16 ± 3
Glutamate	898 ± 151	1216 ± 87	1141 ± 53	1059 ± 60	1001 ± 61	1012 ± 25	1006 ± 94	676 ± 120	860 ± 101	1151 ±82	757 ±128	927 ± 129
Glutamine	2334 ± 451	3217 ± 238	2161 ± 116	2073 ± 176	2753 ± 216	2637 ± 174	2482 ± 295	1949 ± 337	1937 ± 295	2016 ±139	4211 ± 935	2289 ± 417
Glycine	753 ± 127	986 ± 64	810 ± 32	791 ± 40	795 ± 67	815 ± 46	989 ± 88	667 ± 114	897 ± 108	996 ± 78	947 ± 143	934 ± 116
Histidine	173 ± 28	197 ± 13	153 ± 14	192 ± 14	318 ± 24	188 ± 12	250 ± 22	159 ± 21	156 ± 19	261 ± 17	385 ± 64	232 ± 30
Isoleucine	494 ± 85	570 ± 42	413 ± 32	406 ± 30	505 ± 41	541 ± 44	695 ± 68	430 ± 67	536 ± 70	602 ± 59	539 ± 88	635 ± 92
Leucine	620 ± 108	740 ± 56	504 ± 45	531 ± 41	612 ± 70	642 ± 61	938 ± 97	575 ± 92	672 ± 97	833 ± 81	787 ± 165	783 ± 124
Lysine	884 ± 172	898 ± 57	788 ± 50	747 ± 53	718 ± 60	857 ± 67	940 ± 107	545 ± 99	815 ± 132	902 ± 94	797 ± 180	936 ± 150
Methionine	92 ± 15	122± 6	94 ± 4	90 ± 4	86 ± 4	94 ± 4	128 ± 13	103 ± 17	132 ± 17	132 ± 14	111 ± 20	125 ± 5
Phenylalanine	466 ± 74	543 ± 25	461 ± 30	435 ± 27	546 ± 36	511 ± 43	596 ± 50	395 ± 55	464 ± 50	521 ± 34	555 ± 64	511± 62
Proline	2269 ± 373	2493 ± 160	2501 ± 168	2336 ± 120	3429 ±258	2593 ± 270	2678 ± 306	1697 ± 301	2088 ± 291	2657 ± 182	2382 ± 331	2382 ± 324
Serine	4017 ± 683	4458 ± 361	4796 ± 113	4020 ± 255	4374 ± 345	4515 ± 315	4537 ± 484	2985 ± 578	3687 ± 503	4926 ± 651	2970 ± 582	4563 ± 729
Threonine	332 ± 58	393 ± 29	352 ± 18	329 ± 27	436 ± 34	385 ± 30	444 ± 58	289 ± 46	387 ± 48	435 ± 37	529 ± 82	417 ± 61
Tryptophan	289 ± 41	311 ± 7	281 ± 12	263 ± 9	297 ± 15	318 ± 16	366 ± 21	257 ± 30	315 ± 29	336 ± 20	328 ± 39	336 ± 39
Tyrosine	1006 ± 171	2115 ± 186	2102 ± 189	1980 ± 122	1783 ± 168	2142 ± 126	1775 ± 229	667 ± 91	929 ± 106	1094 ± 99	1881 ± 273	1440 ± 274
Valine	538 ± 90	711 ± 66	407 ± 28	496 ± 33	634± 62	537 ± 41	689 ± 74	470 ± 83	458 ± 63	604 ± 47	723 ± 141	565 ± 88

*n = 4
